# Supplementary material for: Access to care solutions in healthcare for obstetric care in Africa: A systematic review
Source: PLoS One. 2021 Jun 4;16(6):e0252583. doi: 10.1371/journal.pone.0252583 (PMC8177460; doi:10.1371/journal.pone.0252583)
Supplement: S1 Table — (DOCX) [file pone.0252583.s001.docx]

Supplemental table 1: Search Strategy

| PubMed/MEDLINE | | |
| --- | --- | --- |
| Set | Terms | Results |
| #1 | prehospital[tiab] OR "pre-hospital"[tiab] | 14287 |
| #2  Transportation methods or prehospital care | "Transportation"[Mesh] OR "Transportation of Patients"[Mesh] OR transportation[tiab] OR transported[tiab] OR transporting[tiab] OR transports[tiab] OR ambulance[tiab] OR ambulances[tiab] OR vehicle[tiab] OR auto[tiab] OR automobile[tiab] OR automobiles[tiab] OR car[tiab] OR cars[tiab] OR taxi[tiab] OR taxis[tiab] OR bus[tiab] OR autobus[tiab] OR subway[tiab] OR trains[tiab] OR bicycle[tiab] OR bicycles[tiab] OR motorcycle[tiab] OR motorcycles[tiab] OR motorbike[tiab] OR motorbikes[tiab] OR bike[tiab] OR bikes[tiab] OR kombi[tiab] OR rickshaw[tiab] OR rickshaws[tiab] OR "tuk tuk"[tiab] OR ((travel*[tiab] OR transport*[tiab]) AND ("Economics"[Mesh] OR economics[tiab] OR economic[tiab] OR voucher[tiab] OR vouchers[tiab] OR loan[tiab] OR loans[tiab] OR fund[tiab] OR funded[tiab] OR funds[tiab] OR funding[tiab] OR financ*[tiab])) | 334326 |
| #3  Health service – emergency, maternal, follow up | "Emergency Medical Services"[Mesh:NoExp] OR "Emergency Treatment"[Mesh:NoExp] OR "Advanced Trauma Life Support Care"[Mesh] OR "First Aid"[Mesh] OR "Resuscitation"[Mesh] OR "Emergency Service, Hospital"[Mesh] OR "Health Facilities"[Mesh] OR "Referral and Consultation"[Mesh] OR "Case Management"[Mesh] OR emergency[tiab] OR casualty[tiab] OR resuscitat*[tiab] OR "first aid"[tiab] OR "life support"[tiab] OR "health center*"[tiab] OR "health department*"[tiab] OR rehabilitation[tiab] OR clinic[tiab] OR clinics[tiab] OR hospital[tiab] OR hospitals[tiab] OR referral[tiab] OR ("follow up"[tiab] AND (visit[tiab] OR visits[tiab] OR appointment[tiab] OR appointments[tiab])) | 2151074 |
| #4 | (#2 AND #3) | 43068 |
| #5 | #1 OR #4 | 53150 |
| #6  Trauma or maternity patients | "Accidents"[Mesh] OR "Wounds and Injuries"[Mesh] OR "injuries"[sh] OR "Suicide"[Mesh] OR "Self-Injurious Behavior"[Mesh] OR "Violence"[Mesh] OR "Bites and Stings"[Mesh] OR "Burns"[Mesh] OR "Drowning"[Mesh] OR "Poisoning"[Mesh] OR "poisoning"[sh] OR "Maternal Health Services"[Mesh] OR "Labor, Obstetric"[Mesh] OR "Delivery, Obstetric"[Mesh] OR "Pregnancy"[Mesh] OR "Pregnant Women"[Mesh] OR "Pregnancy Complications"[Mesh] OR Accident[tiab] OR accidents[tiab] OR accidental[tiab] OR crash[tiab] OR wound[tiab] OR wounds[tiab] OR injury[tiab] OR injured[tiab] OR injuries[tiab] OR injurious[tiab] OR suicide[tiab] OR suicides[tiab] OR suicidal[tiab] OR hanging[tiab] OR hung[tiab] OR violence[tiab] OR violent[tiab] OR assault[tiab] OR assaults[tiab] OR contusion[tiab] OR contusions[tiab] OR bite[tiab] OR bites[tiab] OR snakebite[tiab] OR snakebites[tiab] OR envenomation[tiab] OR envenomations[tiab] OR venom[tiab] OR bitten[tiab] OR sting[tiab] OR stings[tiab] OR stung[tiab] OR gunshot[tiab] OR "gun shot"[tiab] OR burn[tiab] OR burns[tiab] OR burned[tiab] OR burnt[tiab] OR drowning[tiab] OR drownings[tiab] OR drowned[tiab] OR electrocution[tiab] OR electrocutions[tiab] OR maternal[tiab] OR prenatal[tiab] OR prenatal[tiab] OR pre-natal[tiab] OR postnatal[tiab] OR post-natal[tiab] OR birth[tiab] OR perinatal[tiab] OR peri-natal[tiab] OR obstetric[tiab] | 3144218 |
| #7  Africa | "Africa"[Mesh] OR Africa[all fields] OR Algeria[all fields] OR Angola[all fields] OR Benin[all fields] OR Botswana[all fields] OR "Burkina Faso"[all fields] OR Burundi[all fields] OR "Cabo Verde"[all fields] OR "Cape Verde"[all fields] OR "Central African"[all fields] OR Chad[all fields] OR Comoros[all fields] OR Congo[all fields] OR "Cote d Ivoire"[all fields] OR "Cote dIvoire"[all fields] OR Congo[all fields] OR Djibouti[all fields] OR Egypt[all fields] OR "Guinea"[all fields] OR Eritrea[all fields] OR Ethiopia[all fields] OR Gabon[all fields] OR Gambia[all fields] OR Ghana[all fields] OR Guinea[all fields] OR Kenya[all fields] OR Lesotho[all fields] OR Liberia[all fields] OR Libya[all fields] OR Libyan[all fields] OR Madagascar[all fields] OR Malawi[all fields] OR Mali[all fields] OR Mauritania[all fields] OR Mayotte[all fields] OR Morocco[all fields] OR Mozambique[all fields] OR Namibia[all fields] OR Niger[all fields] OR Nigeria[all fields] OR Rwanda[all fields] OR Sahel[all fields] OR "Sao Tome and Principe"[all fields] OR Senegal[all fields] OR "Sierra Leone"[all fields] OR Somalia[all fields] OR "South Africa"[all fields] OR "South Sudan"[all fields] OR Sudan[all fields] OR Swaziland[all fields] OR Tanzania[all fields] OR Togo[all fields] OR Tunisia[all fields] OR Uganda[all fields] OR Sahara[all fields] OR Zambia[all fields] OR Zimbabwe[all fields] | 643627 |
| #8 | #5 AND #6 AND #7 | 1139 |
| Embase | | |
| Set | Terms | Results |
| #1 | prehospital:ab,ti OR "pre-hospital":ab,ti | 20104 |
| #2  Transportation methods or prehospital care | 'traffic and transport'/exp OR 'patient transport'/exp OR transportation:ab,ti OR transported:ab,ti OR transporting:ab,ti OR transports:ab,ti OR ambulance:ab,ti OR ambulances:ab,ti OR vehicle:ab,ti OR auto:ab,ti OR automobile:ab,ti OR automobiles:ab,ti OR car:ab,ti OR cars:ab,ti OR taxi:ab,ti OR taxis:ab,ti OR bus:ab,ti OR autobus:ab,ti OR subway:ab,ti OR trains:ab,ti OR bicycle:ab,ti OR bicycles:ab,ti OR motorcycle:ab,ti OR motorcycles:ab,ti OR motorbike:ab,ti OR motorbikes:ab,ti OR bike:ab,ti OR bikes:ab,ti OR kombi:ab,ti OR rickshaw:ab,ti OR rickshaws:ab,ti OR "tuk tuk":ab,ti OR ((travel*:ab,ti OR transport*:ab,ti) AND ('economics'/exp OR economics:ab,ti OR economic:ab,ti OR voucher:ab,ti OR vouchers:ab,ti OR loan:ab,ti OR loans:ab,ti OR fund:ab,ti OR funded:ab,ti OR funds:ab,ti OR funding:ab,ti OR financ*:ab,ti)) | 544949 |
| #3  Health service – emergency, maternal, follow up | 'emergency health service'/exp OR 'emergency treatment'/exp OR 'health care facility'/exp OR 'patient referral'/exp OR 'case management'/exp OR emergency:ab,ti OR casualty:ab,ti OR resuscitat*:ab,ti OR "first aid":ab,ti OR "life support":ab,ti OR "health center*":ab,ti OR "health department*":ab,ti OR rehabilitation:ab,ti OR clinic:ab,ti OR clinics:ab,ti OR hospital:ab,ti OR hospitals:ab,ti OR referral:ab,ti OR ("follow up":ab,ti AND (visit:ab,ti OR visits:ab,ti OR appointment:ab,ti OR appointments:ab,ti)) | 3377419 |
| #4 | (#2 AND #3) | 93219 |
| #5 | #1 OR #4 | 106918 |
| #6  Trauma or maternity patients | 'accident'/exp OR 'injury'/exp OR 'suicide'/exp OR 'violence'/exp OR 'bites and stings'/exp OR 'burn'/exp OR 'drowning'/exp OR 'intoxication'/exp OR 'maternal care'/exp OR 'pregnancy disorder'/exp OR 'pregnancy'/exp OR 'obstetric delivery'/exp OR Accident:ab,ti OR accidents:ab,ti OR accidental:ab,ti OR crash:ab,ti OR wound:ab,ti OR wounds:ab,ti OR injury:ab,ti OR injured:ab,ti OR injuries:ab,ti OR injurious:ab,ti OR suicide:ab,ti OR suicides:ab,ti OR suicidal:ab,ti OR hanging:ab,ti OR hung:ab,ti OR violence:ab,ti OR violent:ab,ti OR assault:ab,ti OR assaults:ab,ti OR contusion:ab,ti OR contusions:ab,ti OR bite:ab,ti OR bites:ab,ti OR snakebite:ab,ti OR snakebites:ab,ti OR envenomation:ab,ti OR envenomations:ab,ti OR venom:ab,ti OR bitten:ab,ti OR sting:ab,ti OR stings:ab,ti OR stung:ab,ti OR gunshot:ab,ti OR "gun shot":ab,ti OR burn:ab,ti OR burns:ab,ti OR burned:ab,ti OR burnt:ab,ti OR drowning:ab,ti OR drownings:ab,ti OR drowned:ab,ti OR electrocution:ab,ti OR electrocutions:ab,ti OR maternal:ab,ti OR prenatal:ab,ti OR prenatal:ab,ti OR pre-natal:ab,ti OR postnatal:ab,ti OR post-natal:ab,ti OR birth:ab,ti OR perinatal:ab,ti OR peri-natal:ab,ti OR obstetric:ab,ti | 4751929 |
| #7  Africa | 'Africa'/exp OR Africa:ab,ti,ca OR Algeria:ab,ti,ca OR Angola:ab,ti,ca OR Benin:ab,ti,ca OR Botswana:ab,ti,ca OR "Burkina Faso":ab,ti,ca OR Burundi:ab,ti,ca OR "Cabo Verde":ab,ti,ca OR "Cape Verde":ab,ti,ca OR "Central African":ab,ti,ca OR Chad:ab,ti,ca OR Comoros:ab,ti,ca OR Congo:ab,ti,ca OR "Cote d Ivoire":ab,ti,ca OR "Cote dIvoire":ab,ti,ca OR Congo:ab,ti,ca OR Djibouti:ab,ti,ca OR Egypt:ab,ti,ca OR "Equatorial Guinea":ab,ti,ca OR Eritrea:ab,ti,ca OR Ethiopia:ab,ti,ca OR Gabon:ab,ti,ca OR Gambia:ab,ti,ca OR Ghana:ab,ti,ca OR Guinea:ab,ti,ca OR Kenya:ab,ti,ca OR Lesotho:ab,ti,ca OR Liberia:ab,ti,ca OR Libya:ab,ti,ca OR Libyan:ab,ti,ca OR Madagascar:ab,ti,ca OR Malawi:ab,ti,ca OR Mali:ab,ti,ca OR Mauritania:ab,ti,ca OR Mayotte:ab,ti,ca OR Morocco:ab,ti,ca OR Mozambique:ab,ti,ca OR Namibia:ab,ti,ca OR Niger:ab,ti,ca OR Nigeria:ab,ti,ca OR Rwanda:ab,ti,ca OR Sahel:ab,ti,ca OR "Sao Tome and Principe":ab,ti,ca OR Senegal:ab,ti,ca OR "Sierra Leone":ab,ti,ca OR Somalia:ab,ti,ca OR "South Africa":ab,ti,ca OR "South Sudan":ab,ti,ca OR Sudan:ab,ti,ca OR Swaziland:ab,ti,ca OR Tanzania:ab,ti,ca OR Togo:ab,ti,ca OR Tunisia:ab,ti,ca OR Uganda:ab,ti,ca OR Sahara:ab,ti,ca OR Zambia:ab,ti,ca OR Zimbabwe:ab,ti,ca | 728121 |
| #8 | #5 AND #6 AND #7 | 2076 |
| #9 | #8 AND [embase]/lim NOT [medline]/lim | 784 |
| CINAHL | | |
| Set | Terms | Results |
| #1 | TI ( prehospital OR "pre-hospital" ) OR AB ( prehospital OR "pre-hospital" ) | 7765 |
| #2  Transportation methods or prehospital care | (MH "Transportation+") OR (MH "Transportation of Patients+") OR TI (transportation OR transported OR transporting OR transports OR ambulance OR ambulances OR vehicle OR auto OR automobile OR automobiles OR car OR cars OR taxi OR taxis OR bus OR autobus OR subway OR trains OR bicycle OR bicycles OR motorcycle OR motorcycles OR motorbike OR motorbikes OR bike OR bikes OR kombi OR rickshaw OR rickshaws OR "tuk tuk" OR ((travel* OR transport*) AND (economics OR economic OR voucher OR vouchers OR loan OR loans OR fund OR funded OR funds OR funding OR financ*))) OR AB (transportation OR transported OR transporting OR transports OR ambulance OR ambulances OR vehicle OR auto OR automobile OR automobiles OR car OR cars OR taxi OR taxis OR bus OR autobus OR subway OR trains OR bicycle OR bicycles OR motorcycle OR motorcycles OR motorbike OR motorbikes OR bike OR bikes OR kombi OR rickshaw OR rickshaws OR "tuk tuk" OR ((travel* OR transport*) AND (economics OR economic OR voucher OR vouchers OR loan OR loans OR fund OR funded OR funds OR funding OR financ*))) | 75056 |
| #3  Health service – emergency, maternal, follow up | (MH "Health Facilities+") OR (MH "Emergency Service+") OR (MH "Emergency Treatment+") OR (MH "Emergency Medical Services+") OR (MH "Case Management") OR (MH "Referral and Consultation+")  OR TI (emergency OR casualty OR resuscitat* OR "first aid" OR "life support" OR "health center*" OR "health department*" OR rehabilitation OR clinic OR clinics OR hospital OR hospitals OR referral OR ("follow up" AND (visit OR visits OR appointment OR appointments))) OR AB (emergency OR casualty OR resuscitat* OR "first aid" OR "life support" OR "health center*" OR "health department*" OR rehabilitation OR clinic OR clinics OR hospital OR hospitals OR referral OR ("follow up" AND (visit OR visits OR appointment OR appointments))) | 876245 |
| #4 | (#2 AND #3) | 26346 |
| #5 | #1 OR #4 | 31751 |
| #6  Trauma or maternity patients | (MH "Accidents+") OR (MH "Wounds and Injuries+") OR (MH "Suicide+") OR (MH "Self-Injurious Behavior") OR (MH "Bites and Stings+") OR (MH "Violence+") OR (MH "Poisoning+") OR (MH "Obstetric Emergencies") OR (MH "Obstetric Patients") OR (MH "Maternal-Child Care+") OR (MH "Pregnancy+") OR (MH "Pregnancy, Multiple+") OR (MH "Postnatal Period+") OR (MH "Pregnancy Complications+") OR TI (Accident OR accidents OR accidental OR crash OR wound OR wounds OR injury OR injured OR injuries OR injurious OR suicide OR suicides OR suicidal OR hanging OR hung OR violence OR violent OR assault OR assaults OR contusion OR contusions OR bite OR bites OR snakebite OR snakebites OR envenomation OR envenomations OR venom OR bitten OR sting OR stings OR stung OR gunshot OR "gun shot" OR burn OR burns OR burned OR burnt OR drowning OR drownings OR drowned OR electrocution OR electrocutions OR maternal OR prenatal OR prenatal OR pre-natal OR postnatal OR post-natal OR birth OR perinatal OR peri-natal OR obstetric) OR AB (Accident OR accidents OR accidental OR crash OR wound OR wounds OR injury OR injured OR injuries OR injurious OR suicide OR suicides OR suicidal OR hanging OR hung OR violence OR violent OR assault OR assaults OR contusion OR contusions OR bite OR bites OR snakebite OR snakebites OR envenomation OR envenomations OR venom OR bitten OR sting OR stings OR stung OR gunshot OR "gun shot" OR burn OR burns OR burned OR burnt OR drowning OR drownings OR drowned OR electrocution OR electrocutions OR maternal OR prenatal OR prenatal OR pre-natal OR postnatal OR post-natal OR birth OR perinatal OR peri-natal OR obstetric) | 791132 |
| #7  Africa | MH (Africa) OR TI (Africa OR Algeria OR Angola OR Benin OR Botswana OR "Burkina Faso" OR Burundi OR "Cabo Verde" OR "Cape Verde" OR "Central African" OR Chad OR Comoros OR Congo OR "Cote d Ivoire" OR "Cote dIvoire" OR Congo OR Djibouti OR Egypt OR "Guinea" OR Eritrea OR Ethiopia OR Gabon OR Gambia OR Ghana OR Guinea OR Kenya OR Lesotho OR Liberia OR Libya OR Libyan OR Madagascar OR Malawi OR Mali OR Mauritania OR Mayotte OR Morocco OR Mozambique OR Namibia OR Niger OR Nigeria OR Rwanda OR Sahel OR "Sao Tome and Principe" OR Senegal OR "Sierra Leone" OR Somalia OR "South Africa" OR "South Sudan" OR Sudan OR Swaziland OR Tanzania OR Togo OR Tunisia OR Uganda OR Sahara OR Zambia OR Zimbabwe) OR AB (Africa OR Algeria OR Angola OR Benin OR Botswana OR "Burkina Faso" OR Burundi OR "Cabo Verde" OR "Cape Verde" OR "Central African" OR Chad OR Comoros OR Congo OR "Cote d Ivoire" OR "Cote dIvoire" OR Congo OR Djibouti OR Egypt OR "Guinea" OR Eritrea OR Ethiopia OR Gabon OR Gambia OR Ghana OR Guinea OR Kenya OR Lesotho OR Liberia OR Libya OR Libyan OR Madagascar OR Malawi OR Mali OR Mauritania OR Mayotte OR Morocco OR Mozambique OR Namibia OR Niger OR Nigeria OR Rwanda OR Sahel OR "Sao Tome and Principe" OR Senegal OR "Sierra Leone" OR Somalia OR "South Africa" OR "South Sudan" OR Sudan OR Swaziland OR Tanzania OR Togo OR Tunisia OR Uganda OR Sahara OR Zambia OR Zimbabwe) OR AF (Africa OR Algeria OR Angola OR Benin OR Botswana OR "Burkina Faso" OR Burundi OR "Cabo Verde" OR "Cape Verde" OR "Central African" OR Chad OR Comoros OR Congo OR "Cote d Ivoire" OR "Cote dIvoire" OR Congo OR Djibouti OR Egypt OR "Guinea" OR Eritrea OR Ethiopia OR Gabon OR Gambia OR Ghana OR Guinea OR Kenya OR Lesotho OR Liberia OR Libya OR Libyan OR Madagascar OR Malawi OR Mali OR Mauritania OR Mayotte OR Morocco OR Mozambique OR Namibia OR Niger OR Nigeria OR Rwanda OR Sahel OR "Sao Tome and Principe" OR Senegal OR "Sierra Leone" OR Somalia OR "South Africa" OR "South Sudan" OR Sudan OR Swaziland OR Tanzania OR Togo OR Tunisia OR Uganda OR Sahara OR Zambia OR Zimbabwe) | 87639 |
| #8 | #5 AND #6 AND #7 | 389 |
| Scopus | | |
| Set | Terms | Results |
| #1 | TITLE-ABS-KEY(prehospital OR "pre-hospital") | 17583 |
| #2  Transportation methods or prehospital care | TITLE-ABS-KEY(transportation OR transported OR transporting OR transports OR ambulance OR ambulances OR vehicle OR auto OR automobile OR automobiles OR car OR cars OR taxi OR taxis OR bus OR autobus OR subway OR trains OR bicycle OR bicycles OR motorcycle OR motorcycles OR motorbike OR motorbikes OR bike OR bikes OR kombi OR rickshaw OR rickshaws OR "tuk tuk" OR ((travel* OR transport*) AND (economics OR economic OR voucher OR vouchers OR loan OR loans OR fund OR funded OR funds OR funding OR financ*))) | 3154187 |
| #3  Health service – emergency, maternal, follow up | TITLE-ABS-KEY(emergency OR casualty OR resuscitat* OR "first aid" OR "life support" OR "health center*" OR "health department*" OR rehabilitation OR clinic OR clinics OR hospital OR hospitals OR referral OR ("follow up" AND (visit OR visits OR appointment OR appointments))) | 2934836 |
| #4 | (#2 AND #3) | 107903 |
| #5 | #1 OR #4 | 119276 |
| #6  Trauma or maternity patients | TITLE-ABS-KEY(Accident OR accidents OR accidental OR crash OR wound OR wounds OR injury OR injured OR injuries OR injurious OR suicide OR suicides OR suicidal OR hanging OR hung OR violence OR violent OR assault OR assaults OR contusion OR contusions OR bite OR bites OR snakebite OR snakebites OR envenomation OR envenomations OR venom OR bitten OR sting OR stings OR stung OR gunshot OR "gun shot" OR burn OR burns OR burned OR burnt OR drowning OR drownings OR drowned OR electrocution OR electrocutions OR maternal OR prenatal OR prenatal OR pre-natal OR postnatal OR post-natal OR birth OR perinatal OR peri-natal OR obstetric) | 3761141 |
| #7  Africa | TITLE-ABS-KEY(Africa OR Algeria OR Angola OR Benin OR Botswana OR "Burkina Faso" OR Burundi OR "Cabo Verde" OR "Cape Verde" OR "Central African" OR Chad OR Comoros OR Congo OR "Cote d Ivoire" OR "Cote dIvoire" OR Congo OR Djibouti OR Egypt OR "Guinea" OR Eritrea OR Ethiopia OR Gabon OR Gambia OR Ghana OR Guinea OR Kenya OR Lesotho OR Liberia OR Libya OR Libyan OR Madagascar OR Malawi OR Mali OR Mauritania OR Mayotte OR Morocco OR Mozambique OR Namibia OR Niger OR Nigeria OR Rwanda OR Sahel OR "Sao Tome and Principe" OR Senegal OR "Sierra Leone" OR Somalia OR "South Africa" OR "South Sudan" OR Sudan OR Swaziland OR Tanzania OR Togo OR Tunisia OR Uganda OR Sahara OR Zambia OR Zimbabwe) OR AFFILCOUNTRY(Africa OR Algeria OR Angola OR Benin OR Botswana OR "Burkina Faso" OR Burundi OR "Cabo Verde" OR "Cape Verde" OR "Central African" OR Chad OR Comoros OR Congo OR "Cote d Ivoire" OR "Cote dIvoire" OR Congo OR Djibouti OR Egypt OR "Guinea" OR Eritrea OR Ethiopia OR Gabon OR Gambia OR Ghana OR Guinea OR Kenya OR Lesotho OR Liberia OR Libya OR Libyan OR Madagascar OR Malawi OR Mali OR Mauritania OR Mayotte OR Morocco OR Mozambique OR Namibia OR Niger OR Nigeria OR Rwanda OR Sahel OR "Sao Tome and Principe" OR Senegal OR "Sierra Leone" OR Somalia OR "South Africa" OR "South Sudan" OR Sudan OR Swaziland OR Tanzania OR Togo OR Tunisia OR Uganda OR Sahara OR Zambia OR Zimbabwe) | 1770718 |
| #8 | #5 AND #6 AND #7 | 1847 |
| Global Health Library – African Index Medicus | | |
| Set | Terms | Results |
| #1 | prehospital OR "pre-hospital" OR transport* OR ambulance OR ambulances OR vehicle OR auto OR automobile OR automobiles OR car OR cars OR taxi OR taxis OR bus OR autobus OR subway OR trains OR bicycle OR bicycles OR motorcycle OR motorcycles OR motorbike OR motorbikes OR bike OR bikes OR kombi OR rickshaw OR rickshaws OR "tuk tuk" |  |
| #2  Health service – emergency, maternal, follow up | emergency OR casualty OR resuscitat* OR "first aid" OR "life support" OR "health center*" OR "health department*" OR rehabilitation OR clinic OR clinics OR hospital OR hospitals OR referral OR ("follow up" AND (visit OR visits OR appointment OR appointments)) |  |
| #3  Trauma or maternity patients | Accident OR accidents OR accidental OR crash OR wound OR wounds OR injury OR injured OR injuries OR injurious OR suicide OR suicides OR suicidal OR hanging OR hung OR violence OR violent OR assault OR assaults OR contusion OR contusions OR bite OR bites OR snakebite OR snakebites OR envenomation OR envenomations OR venom OR bitten OR sting OR stings OR stung OR gunshot OR "gun shot" OR burn OR burns OR burned OR burnt OR drowning OR drownings OR drowned OR electrocution OR electrocutions OR maternal OR prenatal OR prenatal OR pre-natal OR postnatal OR post-natal OR birth OR perinatal OR peri-natal OR obstetric |  |
| #4 | #1 AND #2 AND #3 | 81 |
